# Motion detection based on recurrent network dynamics

**DOI:** 10.3389/fnsys.2014.00239

**Published:** 2014-12-23

**Authors:** Jeroen Joukes, Till S. Hartmann, Bart Krekelberg

**Affiliations:** Center for Molecular and Behavioral Neuroscience, Rutgers UniversityNewark, NJ, USA

**Keywords:** motion, model, recurrent connections, time, middle temporal area

## Abstract

The detection of visual motion requires temporal delays to compare current with earlier visual input. Models of motion detection assume that these delays reside in separate classes of slow and fast thalamic cells, or slow and fast synaptic transmission. We used a data-driven modeling approach to generate a model that instead uses recurrent network dynamics with a single, fixed temporal integration window to implement the velocity computation. This model successfully reproduced the temporal response dynamics of a population of motion sensitive neurons in macaque middle temporal area (MT) and its constituent parts matched many of the properties found in the motion processing pathway (e.g., Gabor-like receptive fields (RFs), simple and complex cells, spatially asymmetric excitation and inhibition). Reverse correlation analysis revealed that a simplified network based on first and second order space-time correlations of the recurrent model behaved much like a feedforward motion energy (ME) model. The feedforward model, however, failed to capture the full speed tuning and direction selectivity properties based on higher than second order space-time correlations typically found in MT. These findings support the idea that recurrent network connectivity can create temporal delays to compute velocity. Moreover, the model explains why the motion detection system often behaves like a feedforward ME network, even though the anatomical evidence strongly suggests that this network should be dominated by recurrent feedback.

## Introduction

Successful interaction with a dynamic environment requires a neural mechanism for the detection of motion. In the dominant model of motion perception in the primate—the motion energy (ME) model (Adelson and Bergen, 1985; Watson and Ahumada, 1985; Krekelberg, 2008)—the temporal component that is essential for the detection of motion is implemented as a class of neurons that have slow response dynamics.

The primate visual system does contain a class of slower neurons (the parvocellular stream), but the evidence that they are a critical component in motion detection (Malpeli et al., 1981; Nealey and Maunsell, 1994; DeValois and Cottaris, 1998; De Valois et al., 2000) is controversial. For instance, layer IVCα of the primary visual cortex (V1), contains numerous direction selective (DS) cells, but mainly receives magnocellular input (Blasdel and Fitzpatrick, 1984) and, consistent with this, inactivation of the magnocellular layers of the lateral geniculate nucleus (LGN) disrupts motion processing in the middle temporal area (MT), while inactivation of parvocellular layers has little effect (Maunsell et al., 1990). Hence, even though it is clear that motion sensitive neurons in the primate receive two sets of inputs, one delayed with respect to the other (DeValois and Cottaris, 1998; De Valois et al., 2000; Priebe and Ferster, 2005), the origin of these delays remains unknown.

A biophysically realistic model of motion detection (Maex and Orban, 1996) ascribes the temporal delays to intrinsic differences between slow and fast synaptic transmission. However, such intrinsic differences are fixed, and it is difficult to see how they alone can account for the observed wide range of preferred speeds (see Section Discussion).

Even though anatomically cortical networks are clearly dominated by recurrent connections, this connectivity plays at best a subordinate role in most models of motion detection. For instance, the ME model was originally envisaged as entirely feedforward although it has been extended with recurrent connectivity to amplify direction selectivity (Douglas et al., 1995; Suarez et al., 1995; Maex and Orban, 1996) or to generate motion integration and segmentation (Bayerl and Neumann, 2004; Tlapale et al., 2010). The analytic work of Mineiro and Zipser (1998) and Sabatini and Solari (1999), however, has shown that recurrent connectivity alone is in principle sufficient to generate direction selectivity and (Clifford et al., 1997; Clifford and Langley, 2000) mathematically showed that a recursive implementation of the temporal filter of the ME model can greatly reduce the amount of storage and computation needed for a motion detector tuned to a broad spatiotemporal frequency range.

Our works starts from the data—a set of recordings from MT neurons—and shows that an artificial recurrent neural network can faithfully reproduce the speed and direction tuned responses to visual motion. New insights into motion mechanisms resulted from a detailed, quantitative investigation of this model network. Notably, no separate classes of fast and slow neurons, or carefully tuned delay lines were needed to generate a wide range of speed preferences. Instead, a range of temporal delays and concomitant speed preferences emerged from the weight patterns of the network. Second, while the recurrent network could be approximated by a ME model, such a feedforward approximation failed to capture the sequential recruitment typically found in MT neurons (Mikami, 1992). Finally, the response properties of the units in the recurrent network (e.g., Gabor receptive fields (RFs), simple- and complex-like responses), showed a remarkable match with the known properties of neurons in the motion processing pathway.

## Materials and methods

### Experimental data

#### Subjects

We measured the speed tuning properties in area MT of two adult male rhesus monkeys (*Macaca mulatta*). Experimental and surgical protocols conformed to United States Department of Agriculture regulations and the National Institutes of Health guidelines for humane care and use of laboratory animals and were approved by the local IACUC committee.

#### Visual stimulation

The visual stimuli were generated with in-house OpenGL software (Quadro Pro Graphics card, 1024 × 768 pixels, 8 bits/pixel) and displayed on a 21 inch monitor (75 Hz, non-interlaced, 1024 × 768 pixels; model GDM-2000TC; Sony). Monkeys viewed the stimuli from a distance of 57 cm in a dark room (<0.5 cd/m^2^) while seated in a standard primate chair (Crist Instruments, Germantown, MD, USA) with the head post supported by the chair frame. We sampled eye position at 60 Hz using an infrared system (IScan, Burlington, MA, USA), and monitored and recorded the eye position data with the NIMH Cortex program, which also controlled stimulus presentation.

#### Stimuli and experimental paradigm

We mapped velocity tuning with a random dot pattern that consisted of 100 dots within a 10° diameter circular aperture. The dots had infinite lifetime and were randomly repositioned after leaving the aperture. The dots were 0.15° in diameter and had a luminance of 30 cd/m^2^. Compared with the 5 cd/m^2^ background, this resulted in a Michelson point contrast of 70%.

The activity of single units in area MT was recorded with tungsten microelectrodes (3–5 MOhm; Frederick Haer Company, Bowdoinham, ME, USA), which we inserted using a hydraulic micropositioner (model 650; David Kopf Instruments, Tujunga, CA, USA). We filtered, sorted, and stored the signals using the Plexon (Dallas, TX, USA) system. Area MT was identified by its high proportion of cells with directional selective responses, small RFs relative to those of neighboring medial superior temporal area, and its location on the posterior bank of the superior temporal sulcus. The typical recording depth was in agreement with the expected anatomical location of MT determined by structural magnetic resonance scans.

We determined the directional selectivity and RFs of the cells using automated methods (for details, see Krekelberg and Albright, 2005). Based on the RF center and the preferred direction of motion (rounded to the nearest multiple of 45°) estimated by these methods we optimized stimuli for subsequent measurements. The mean RF eccentricity and SD was 8 ± 4.3° (range of 3–15°). The random dot patterns appeared 250 ms after the monkey started fixating on a central red dot. After moving in the preferred or anti-preferred direction of the neuron for 500 ms, the pattern was extinguished. The range of speeds was 1, 2, 4, 8, 16, 32, and 64°/s. The 14 conditions (7 speeds, 2 directions) were randomly interleaved and repeated between 4 and 21 times. Trials in which eye position deviated from a 2° wide square window centered on the fixation spot were excluded from analysis.

The MT response to the moving stimuli was binned in 13 ms time windows; the frame rate of the monitor used during the experiments (75 Hz). This allowed us to investigate the emergence of the speed tuning and direction selectivity properties at a temporal resolution that matched the (apparent) motion on the monitor.

### Recurrent motion model

#### Elman recurrent neural network

We modeled the neuronal data with an Elman recurrent neural network (Elman, 1990) implemented in the Matlab Neural Network Toolbox (version 4.0.1). The network consisted of units that are considered a crude approximation of a neuron or a group of neurons (Figure 2A). The units were interconnected with adjustable weights simulating synaptic connections with variable strength. Each unit also had an adjustable bias value.

**Figure 1:**
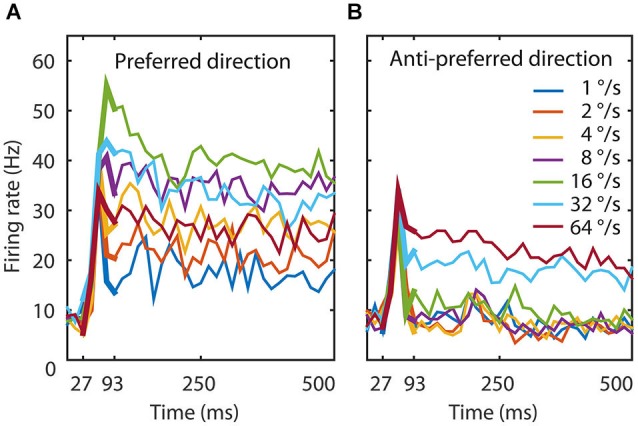
**Experimental data. (A)** The temporal dynamics of the MT population to seven speeds in the preferred direction. The figure shows the average response of 26 MT neurons. The thin lines represent the response to the total duration of the stimulus, the thick lines the time window we used to train the network (27–93 ms). An initial transient lasted 67 ms during which speed tuning and direction selectivity started to emerge. Speed tuning was maximal around 93 ms and followed by a slow reduction in firing rate for most speeds. **(B)** The temporal dynamics of the MT population to seven speeds in the anti-preferred motion direction. Here too, the initial transient lasted 67 ms and direction selectivity was maximal around 93 ms, followed by slow adaptation. The onset response was not strongly direction selective, but after 93 ms the response to the preferred direction could be twice as large as the response to the anti-preferred direction. These data document that the response, speed tuning, and direction tuning change dynamically in the first 100 ms after stimulus presentation.

**Figure 2:**
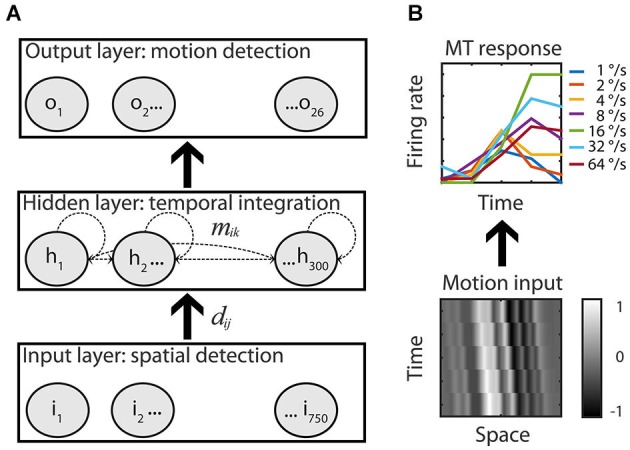
**Recurrent motion model. (A)** An Elman recurrent neural network with 750 input units all-to-all connected to the units of the hidden layer (*d_ij_*). The hidden layer had 300 all-to-all recurrently and laterally connected hidden units (dashed lines, *m_ik_*) that were all-to-all connected to the 26 output units of the RMM. Adjustable weights allowed the network to map motion inputs onto the speed tuned and direction selective response of the 26 recorded MT cells. **(B)** Example input-output. Low-pass filtered white noise patterns (range −1 to 1) shifted with 16°/s in the preferred motion direction over five time bins (bottom) is fitted to the measured response of the example MT cell to the preferred speed in the preferred motion direction (top, solid green line, the six other colors represent the non-preferred speeds).

The network had an input, hidden, and output layer. The input layer consisted of 750 units that simulated a RF of 10° (0.013° per unit); the diameter of the stimulus used during the experiments. The input layer was fully connected to the hidden layer in a feedforward manner. The hidden layer had 300 units that were fully connected to the output layer in a feedforward manner. In addition, all hidden units were laterally/recurrently connected to all hidden units. The output layer consisted of 26 units, each simulating one MT cell. The output for each unit of all layers (indexed by *i*) was calculated by first determining the weighted sum of its inputs plus the bias value: $x_{i}=\sum_{k}w_{ik}y_{k}+b_{i}$, where the index *k* runs over all units that are connected to unit *i*, and then passing this through a sigmoid transfer function: $y_{i}=1/\left(1+e_{i}^{-x}\right)$.

#### Output patterns

We used the model to capture the responses of a representative subset of MT neurons from our sample of 129. To reduce computational complexity, we focused our analyses and modeling on MT cells with robust, DS responses, and band pass speed tuning. The specific criteria for inclusion were the robustness of the response (firing rate > 7 spikes/s averaged over all speeds in the preferred direction), modest to strong direction selectivity (DSI > 0.1, for definition see below), and a preferred speed in the range of 8–32°/s. This selection resulted in a population of 26 MT cells.

The population response revealed an initial response latency of approximately 30 ms followed by the rapid onset of speed and direction tuning that lasted around 100 ms, and finally a sustained phase with relatively constant responses and tuning (Figure 1, thin lines). We chose to train the network on the generation of speed tuning and direction selectivity only (27–93 ms; Figure 1, thick lines). Given the temporal binning in 13 ms time bins (the duration of a monitor frame in the experiment), this resulted in output pattern sequences of firing rates at five time points for each of the 14 conditions (7 speeds, 2 directions) and each of the 26 output units. We normalized the response to a suitable range for the network with a division by the maximum firing rate over all time bins, speeds, directions and MT cells.

#### Input patterns

We recorded responses to preferred and anti-preferred directions of motion only and, therefore, did not attempt to model the entire two-dimensional random dot patterns. Instead, we represented the input as white noise patterns and trained the network to respond in a tuned manner to each of these patterns (Figure 2B). To create the input patterns, 750 (the number of input units) values were randomly assigned a value multiplied with a constant to ensure that the final input values were almost all (4 SD) between −1 and 1. These noise values were spatially low pass filtered by convolving with a Gaussian (*σ* = 0.25°) and a multiplication with a Gaussian envelope over the whole input space (*σ* = 2.5°) to reflect the spatial limits of the RF.

A moving input pattern sequence was modeled by shifting the input pattern in the preferred or anti-preferred direction with one of seven speeds. In the physiological experiments, the visual pattern moved between 0.013° and 0.85° per monitor frame (1°/s–64°/s, respectively). In the model this was implemented by shifting the input pattern by 1–64 input units per 13 ms, respectively.

#### Training phase

Before training the network, we initialized the weights and bias values of all layers with the Nguyen-Widrow algorithm. We trained the recurrent neural network on the input and output pattern sequences we described above in the following way. First, we randomly chose one of seven speeds and a direction of motion. Second, frame-by-frame, a new input pattern sequence for that speed and direction was presented on the input units. Third, for each frame, we calculated the response of the hidden units based on the current feedforward input and the recurrent feedback, and then calculated the response of the output units. Fourth, the error of the network was defined as the difference between the response of the output units and the response of all 26 MT cells (for that speed and direction, and in the corresponding time bin after stimulus onset). This error was used to modify all connection weights in the network using error back-propagation-through-time. We repeated these steps (epochs) five million times until the network converged to reproduce the response of all 26 MT cells. Network parameters were then frozen and we investigated the trained network.

#### Reverse correlation

We probed the neurons of the recurrent motion model (RMM) using reverse correlation analysis. The reverse correlation analysis assumes that the system under study can be described by a set of linear space-time filters followed by a static nonlinearity (linear-nonlinear, or LN model). Even though the LN model is a considerable oversimplification of area MT (and the RMM), we have previously shown that this method can successfully generate quantitative descriptions of receptive field properties in area MT (Hartmann et al., 2011; Richert et al., 2013).

The spike counts needed in this reverse correlation analysis were derived from the RMM activity by scaling the peak response of each unit to 30 spikes per time bin, and then rounding the activity in each bin to the nearest integer. The noise inputs for the reverse correlation analysis were identical to the individual frames of the moving spatial patterns described previously. To reduce computational complexity we used stimuli consisting of 0.027° wide bars for the output units and 0.04° wide bars for the hidden units. This reduced the spatial dimension by a factor of two and three, respectively. The reverse correlation history—the number of time bins leading up to the output activity—was set to be 67 ms. This corresponds to the time needed for the MT population to create a stable speed tuned and DS output. Two million noise stimuli were presented to the model network for reverse correlation analysis of the output units and one million for the hidden units.

We followed standard procedures to estimate the parameters of the LN—model. First, we estimated the spike-triggered average (STA) and spike-triggered covariance (STC) as described in detail in Chichilnisky (2001), Rust et al. (2004) and Simoncelli et al. (2004). Because the STA and STC are not orthogonal filters, we then used the method of Pillow and Simoncelli (2006) to estimate three quantities. First, we estimated the most informative filters in the space spanned by both the STA and STC. These filters are called iSTAC filters (Information theoretic generalization of Spike Triggered Average and Covariance). By construction, these filters best capture the relation between the first and second order statistics of the stimuli and spikes. Second, we estimated the information about the stimulus carried by each of the filters. This quantifies how much a certain filter contributes to the (selective) response of the unit and we use it to rank the filters in order of decreasing importance. Third, we calculated the nonlinearity associated with each filter (for stimuli up to 4 standard deviations away from the mean stimulus). These nonlinearities relate the inner product of a stimulus pattern and the filter (the projection value) to the resulting output of the unit. Examples are shown in Figure 4F. The mathematical details of these computations are described by Pillow and Simoncelli (2006).

**Figure 3:**
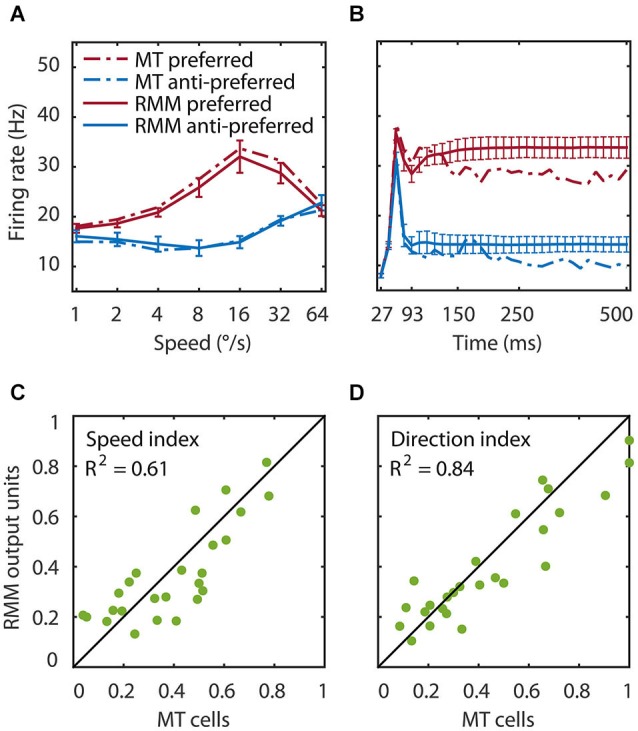
**Speed tuning and direction selectivity of the MT cells and RMM output units**. We simulated the response of the RMM output units to 1000 new patterns and compared their response to the measured MT response. **(A)** The average response over the first 93 ms after stimulus onset. The response is shown for each of seven speeds in the preferred (red) and anti-preferred (blue) motion direction. The dotted lines represent the MT population response, the solid lines the population output of the RMM. The error bars indicate 1 standard deviation over trials. **(B)** The time course of the response averaged over the seven speeds for the trained number of time bins (27–93 ms) and for the full 500 ms of the experiment. **(C,D)** The speed tuning index **(C)** and direction selectivity index **(D)** for the output units (*x*-axis) and the MT cells (*y*-axis). This figure shows that the RMM faithfully captured the temporal dynamics (except for the short term adaptation) as well as the speed tuning and direction selectivity of both the MT population and the single cell response.

**Figure 4:**
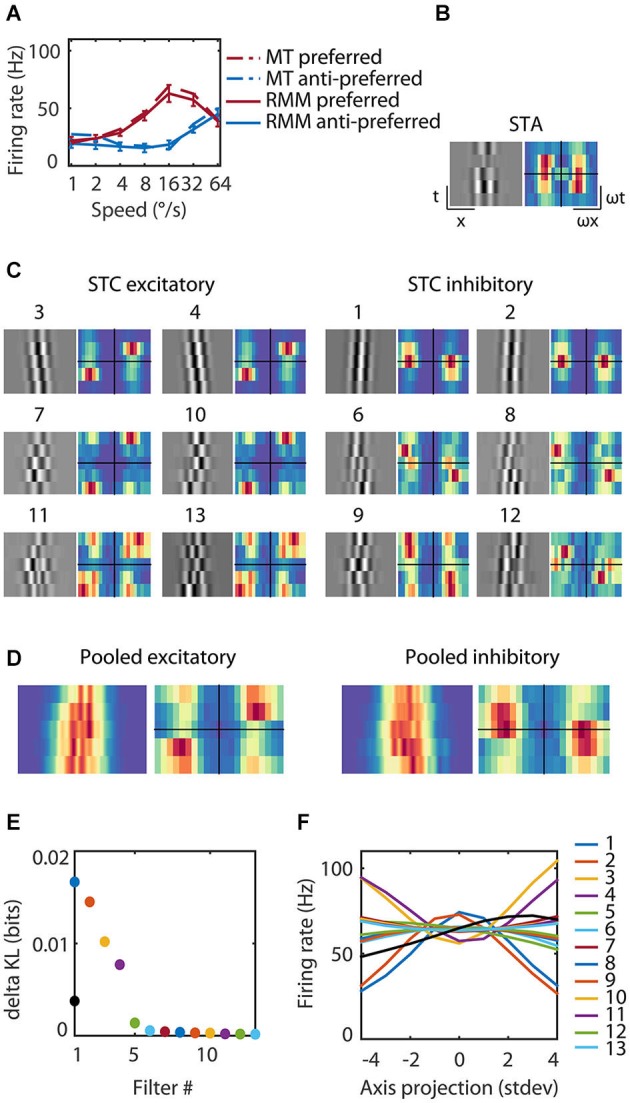
**Reverse correlation analysis of the RMM output units. (A)** The average response over the first 93 ms after stimulus onset for an example MT cell/output unit. The response is shown for each of seven speeds in the preferred (red) and anti-preferred (blue) motion direction. The dotted lines represent the example MT single cell response, the solid lines the example output unit of the RMM. The error bars indicate 1 standard deviation over trials. **(C)** The 13 filters of the RMM ordered by the amount of information (numbers above the filters show rank order). The STA displayed in **(B)** had no clear space time structure as expected for a polarity insensitive output unit and was almost identical to the fifth most informative filter (data not shown). The six excitatory filters (STC Excitatory) had a rightward slant; they were tuned to the preferred speed and direction, with increasing spatial frequencies per quadrature pair. The six suppressive filters (STC Suppressive) had a leftward slant; they were tuned to the anti-preferred direction, with increasing spatial frequencies per quadrature pair. The Fourier spectra are shown next to the 13 filters. For the excitatory filters power was concentrated around the preferred speed and direction of the output unit (16°/s). The Fourier spectra of the suppressive filters had a wider distribution of power over temporal frequency, ranging from stationary to the preferred temporal frequency in the anti-preferred direction and to fast speeds in the preferred direction. **(D)** Pooled excitatory and suppressive filters and Fourier spectra. The pooled excitatory and suppressive filters show that the individual filters largely overlap. The pooled excitatory and suppressive Fourier spectra exemplify the asymmetry of excitation and suppression. **(E)** The amount of information (in bits) contained within the STA (black), the excitatory filters and suppressive filters (see legend panel **(F)**). **(F)** The nonlinearities of the 13 filters. Stimuli that matched a filter (high positive axis projection) or that matched the polarity inverse (high negative axis projection) resulted in a high firing rate for the excitatory filters and a low firing rate for the suppressive filters.

We estimated the speed tuning and direction selectivity properties of the LN-model with 1000 new moving input pattern sequences. For each pattern and for each time bin, the inner product between the motion inputs and the filters gave us the projection value. Ideally, one would calculate the firing rate of the full filter model by passing the projections through a high-dimensional nonlinearity. Due to computer memory constraints, however, we had to restrict this to 1-dimensional nonlinearities; i.e., we assumed that the filter dimensions were separable. The firing rate for the combined filter output was determined as the sum of the individual filter outputs minus the mean for *n*−1 filters.

#### Speed tuning and direction selectivity

For the RMM output and hidden units, the direction selectivity and speed tuning was based on the mean response over time to 1000 new motion inputs for all speeds and directions. For the MT cells we used the mean response over time in all experimental trials. We calculated the direction selectivity index (DSI) with the maximum (average) response over the seven speeds in the preferred direction and the (average) response at that speed in the anti-preferred direction: (preferred−anti-preferred) / (preferred + anti-preferred). For the speed tuning index (SI) we used the maximum and the minimum response across the seven speeds in the preferred direction: (maximum−minimum) / (maximum + minimum).

#### Relative response modulation

We classified the hidden units of the RMM as simple or complex based on their response to sinusoidal gratings with the preferred spatial frequency (0.5 cycles/°) and speed (16°/s) moving in the preferred direction of the unit. After presenting the gratings for 10 s, we removed the response to the first 67 ms (initial transient), and then determined the ratio of the response at the grating temporal frequency (*F*_1_) to the mean response (*F*_0_), averaged over time. The hidden units were classified as simple when *F*_1_/*F*_0_ > 1, complex otherwise (Movshon et al., 1978b; Skottun et al., 1991).

#### Direct and indirect input

We defined a hidden unit’s direct input as weights between the unit and the input units: *d_ij_* represents the strength of the connection from the *j*-th input unit to the *i*-th hidden unit. The rows of matrix ***D*** with entries *d_ij_* therefore represent the direct inputs of each of the hidden units (see Figure 2B). In a recurrent model, however, the units are also modulated by input that travels via one or more other hidden units—we refer to this as the indirect input. In general this indirect input depends on the potentially complex network dynamics, but as a first approximation of this indirect input, we considered only the indirect input that arrives 13 ms (one simulation time step) later than the direct input. We quantified this indirect input by multiplying the connection strength from hidden unit *k* to *i* (*m_ik_*) with the direct input of unit *k* (*d_kj_*), and summing over all hidden units *k*. In matrix notation this simplifies to: ***I*** = ***M*** * ***D***, where ***M*** is the matrix with entries *m*_ik_ (the lateral connections), and ***I*** is the matrix whose rows represent the indirect input.

For each hidden unit, we determined the spatial shift between direct and indirect input as follows. First, we low-passed filtered both the direct and indirect inputs using the same filter used for the motion inputs (see above). Then we calculated the cross-correlation between these two signals and defined the spatial shift (d*x*) as the lag at which the cross-correlation had the largest value. Units where the maximum cross-correlation was below 0.5 (10% of hidden units) were not used in this analysis (Typically these units had weight patterns without coarse spatial structure).

## Results

### Experimental data

We used the velocity tuning curves of 26 MT neurons recorded in two awake macaque monkeys. These recordings were subsets of the data in previously published studies focusing on the influence of contrast Krekelberg Contrast paper 2006, adaptation (Krekelberg et al., 2006), and transparency (Krekelberg and van Wezel, 2013) on speed tuning in MT. The selection criteria for inclusion in this modeling study are described in the Section Materials and Methods.

The population average response to seven speeds in both the preferred and anti-preferred motion direction had an onset delay of 30 ms and an initial transient lasting 70 ms during which speed tuning and direction selectivity started to emerge (Figure 1). Speed tuning and direction selectivity were maximal and stable 90 ms after stimulus onset; although there was a slight overall reduction in firing rate over the remaining 500 ms recorded data. This was likely an effect of adaptation, as has previously been reported in area MT (Kohn and Movshon, 2003; Krekelberg et al., 2006; Schlack et al., 2007).

### Model training

We first investigated whether a network consisting of (artificial) neurons, all with identical intrinsic properties but modifiable synaptic strengths, could reproduce the temporal dynamics and polarity insensitive velocity tuning of the 26 MT cells. Second, we probed this network to determine how its constituent units and connections solved the complex task of motion processing and how its properties related to neurons in the motion processing pathway.

We created a recurrent neural network with 750 input units, 300 recurrently connected hidden units, and 26 output units (Figure 2A, see Section Materials and Methods). The visual input patterns were modeled as one-dimensional random dot patterns moving leftward or rightward at one of seven speeds (Figure 2B, example motion input). The output units were then trained using back-propagation-through-time to reproduce the response of the 26 MT cells when presented with any of the input patterns (see Section Materials and Methods). In the remainder of the results section we highlight salient properties of the model. First, we show that the network reproduced the MT responses; this is a proof of principle that a recurrently connected network whose units all have identical intrinsic properties, could underlie the MT responses. Second, we demonstrate that the output units behaved very much like a ME detector, while also showing that such a feedforward description does not capture the typical time course of direction and speed selectivity. Third, we illustrate how the range of speed tuning in the output units was created from the hidden units. Finally, we present an analysis of the properties of the hidden units that shows how temporal delays and spatial offsets emerged in the network and resulted in the computation of the speed and direction of motion.

### Proof of principle

We tested the performance of the RMM with a simulation of 1000 new input patterns moving at seven speeds in the preferred and anti-preferred motion direction. The average response over time compares the speed tuning properties of the MT and the RMM population response (Figure 3A). The average over speed compares the temporal dynamics over the trained time bins (Figure 3B, thick lines) and over the full 500 ms stimulus presentation time of the experiments (Figure 3B, thin lines). To determine how well individual output units of the RMM captured the properties of the corresponding MT neurons, we calculated the SI (Figure 3C) and DSI (Figure 3D) for both the MT cells and the output units of the RMM (see Section Materials and Methods). As Figure 3 shows, the RMM captured the speed tuning and direction selectivity properties of the individual MT cells over the trained number of time bins and generalized well (i.e., remained in the correct stable state) to the presentation of longer motion stimuli, with low variability across trials (i.e., stimulus patterns).

While the generalization to a new set of random dot patterns demonstrates a degree of robustness and pattern invariance of motion detection in the RMM, a more stringent test is to consider directional selectivity for patterns that were qualitatively different from the (random dot) patterns used in the training procedure. We therefore determined the velocity curves in response to drifting sine wave gratings (SF = 0.5°), and found that these were highly correlated with the velocity curves measured with random dot patterns (*R*^2^ = 0.95). This shows that the RMM was a robust motion detector with a high degree of pattern invariance, consistent with the known properties of area MT (Albright, 1984).

### Comparison with the motion energy model

A common problem in neural network modeling is that one can rarely point at individual elements of the network model as being responsible for a specific component of the input-output transformation. The reason for this is that information and computation are inherently distributed across many elements. This is the same problem experimentalists face when they investigate the motion processing pathway in the real brain. One approach that provides a lower-dimensional description of a complex system uses noise stimuli together with reverse correlation analysis (Chichilnisky, 2001; Rust et al., 2004; Simoncelli et al., 2004). This technique describes a neuron in terms of an equivalent feedforward linear non-linear model by estimating a set of linear filters and their static nonlinearities (LN-model, see Section Materials And Methods). We used this method here to gain insight into the RMM and to allow a direct comparison with the ME model and LN-models based on the response of real neurons to noise stimuli.

We presented visual noise to the RMM and estimated the STA as well as the most informative iSTAC filters and their nonlinearities (See Section Materials and Methods; Pillow and Simoncelli, 2006). Figure 4 shows the estimated LN-model of one of the output units. The spike triggered average (STA, Figure 4B) did not show any clear slanted space time structure nor did it contain much information (STA, Figure 4E). This is expected since the output units were trained to be polarity insensitive, hence little information should be contained in the STA. The 13 most-informative iSTAC filters, however, were clearly slanted in space-time. Six filters were tuned to the preferred direction and six filters were tuned to the anti-preferred direction (Figure 4C). Stimuli that matched a filter (high positive axis projection in Figure 4F) or that matched the polarity inverse of a filter (large negative axis projection in Figure 4F) evoked similar responses. In other words, the nonlinearities were symmetric, hence the output of the filters was polarity insensitive. For the excitatory filters, the unit’s response increased with the match between stimulus and filter. The opposite was true for the anti-preferred filters; the greater the match with the filter, the smaller the response of the output unit. These filters were suppressive. All pairs of preferred and anti-preferred filters were phase-shifted with respect to each other.

If MT neurons were perfect ME detectors, one would predict two pairs of oriented, phase shifted space-time filters, followed by quadratic nonlinearities that evoke excitation from the preferred direction of motion and inhibition from the anti-preferred direction of motion (i.e., opponency). The properties of the first pair of excitatory and the first pair of suppressive filters (first quadruple) qualitatively matched this prediction. However, reverse correlation of this output unit revealed two additional quadruples of filters sensitive to higher spatial frequencies, but overlapping in space-time (Figure 4D). We also note that—unlike the prediction of the ME model—the preferred and anti-preferred filters were not perfectly mirror symmetric. This can be seen most easily in the Fourier spectra (Figure 4C, alongside the filters) and the pooled excitatory and suppressive spectra (Figure 4D). Energy in the preferred filters was concentrated around the preferred speed and direction. The spectrum of the anti-preferred filters, however, was more widely distributed over temporal frequencies (see Section Discussion).

**Figure 5:**
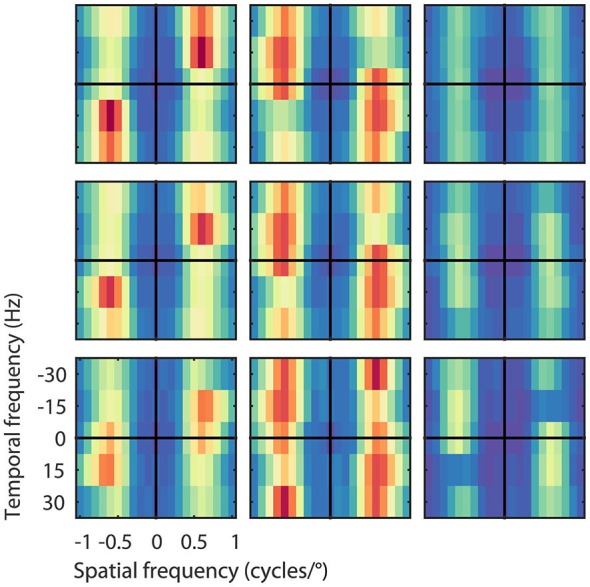
**Velocity computation with asymmetric excitation and suppression**. Pooled excitatory, suppressive, and STA Fourier spectra of the filters (from left to right) for the 26 output units grouped by their preferred speed of 8, 16, and 32°/s (from bottom to top), normalized to the peak power per unit. Excitation was always centered on the preferred speed and direction of the unit, suppression was more broadly distributed across temporal frequencies. As expected, the STA had relatively little motion energy.

The asymmetric spectra from the 26 output units grouped by speed preferences of 8, 16, and 32°/s are shown in Figure 5. As expected, the Fourier spectra of the STA contained almost no ME. The pooled excitatory spectra of the filters were sharply tuned to the preferred speed, while the pooled suppressive spectra were relatively broad. Unlike the prediction of the ME model, the spectra were not mirror copies of each other, which shows that the RMM does not perform a strict subtraction of opposing directions of motion (i.e., motion opponency), but a broader suppressive interaction among multiple Fourier components (Krekelberg and Albright, 2005, motion mechanisms). We emphasize here that the asymmetry of the filters is a feature of the MT data that was successfully captured by the RMM.

### Limitations of feedforward models

Reverse correlation analysis allowed us to reduce the high-dimensional description of the RMM to 13 linear filters and static nonlinearities per output unit. In other words, we determined LN-models that closely matched the input-output relationship of each of the RMM output units. We can now investigate the extent to which these LN-models capture the response to motion. We simulated 1000 motion inputs with seven speeds in both directions and presented them to both the LN-models and the RMM.

**Figure 6:**
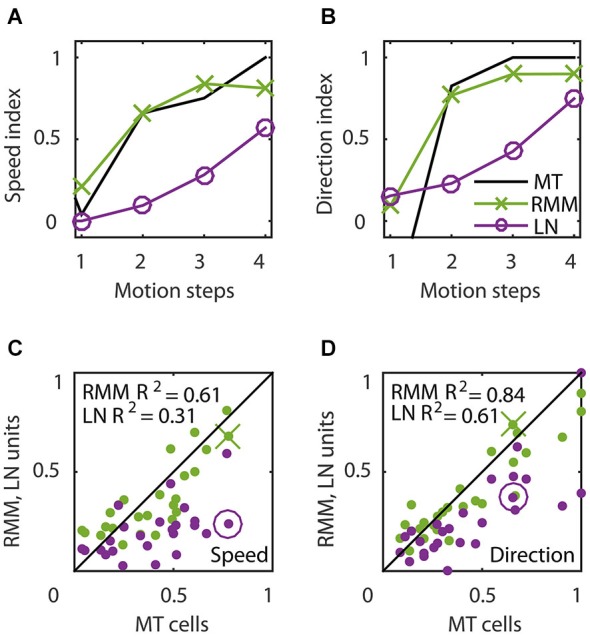
**Temporal dynamics of speed tuning and direction selectivity. (A,B)** Speed tuning index **(A)** and direction selectivity index **(B)** as a function of the number of motion steps for an example MT cell (black curve) and corresponding RMM output unit (green) and LN-model output (magenta). The speed and direction selectivity index of the example RMM and LN unit are displayed in **(C)** and **(D)** with a cross and circle, respectively. **(C)** Average speed tuning index over the motion steps, for all RMM output units (green) and their LN-models (magenta) compared to the speed tuning index of the corresponding MT cells. **(D)** Same as **(C)** for the direction selectivity index. This figure shows that, while the output units of the RMM captured the full speed tuning and direction selectivity properties of most MT cells, the LN model generally fails to do so.

Figure 6 shows the time course of direction selectivity **(A)** and speed tuning **(B)** for an example MT neuron (black curve) over the first four motion steps (time bins 2–5). Both the DSI and SI rise rapidly over the course of the first few time bins of stimulus presentation. Such sequential recruitment has been reported before Mikami (1992). As previously shown in Figure 3, the recurrent model captured this nonlinear behavior quite accurately (green curve). The magenta curve shows the time course of the LN model -the best second-order feedforward approximation to the RMM- for this unit. Clearly, the LN model underestimated the nonlinear response properties. Panels **(C)** and **(D)** in Figure 6 confirm that this was a consistent finding across the sample of 26 output units. For each unit we calculated the DSI and SI per motion step for the MT neuron, and the corresponding RMM and LN model units. Figure 6 compares the average DSI **(C)** and SI **(D)** across the four motion steps between the two models and the MT data. Whereas the output units of the RMM captured most of the speed tuning and direction selectivity properties of the MT cells (*R*^2^ = 0.61 (DSI), *R*^2^ = 0.84 (SI)), the corresponding LN-models performed much worse (*R*^2^ = 0.31 (DSI) and *R*^2^ = 0.61 (SI)). The mismatch between the feedforward model and the MT units was particularly large for MT cells with high direction selectivity and speed tuning.

This finding strongly suggests that an LN-model based only on first and second order space-time correlations was not sufficient to explain the response of single MT cells. Making this claim we need to address two issues. First, the LN-models were based only on the 13 most-informative dimensions; the full LN model contains thousands more dimensions that could collectively describe a considerable amount of information. To address this issue, we note that an LN-model based on filters beyond the 13th filter (up to 100 tested) did not improve speed tuning and direction selectivity compared to the 13 filter LN-model (data not shown). This strongly suggests that the mismatch between the LN model and the data is not due to second-order filters that we excluded from the LN model.

Second, for all LN-models, we summed the output of the individual filters to determine the combined filter output (see Section Materials and Methods) and it is possible that the individual filters should instead be combined nonlinearly to accurately describe the velocity response for the LN-models (Chichilnisky, 2001; Rust et al., 2004; Simoncelli et al., 2004). The course of dimensionality, however, prevents us from accurately estimating the full 13-dimensional nonlinearity. However, we confirmed that the assumption of separability was reasonable by estimating a 4-dimensional nonlinearity for each of the three filter quadruples in Figure 4C (two excitatory and two suppressive filters per spatial frequency) and comparing the predicted firing rate based on the 4-dimensional nonlinearity with the linear summation of the four individual filters per quadruple. Qualitatively, visual inspection of the 4-dimensional nonlinearities did not reveal interactions. This suggests that our separable approximation of the high-dimensional nonlinearity was appropriate. Quantitatively, passing the filter outputs through the 4-dimensional nonlinearities did not improve speed tuning or direction selectivity compared to the separable combination of the filters (data not shown).

Taken together these findings strongly suggest that the MT neurons velocity tuning is sensitive to third and higher order space-time interactions. The RMM captures this sensitivity, but the LN and ME model cannot.

### Speed preferences in the output units

**Figure 7:**
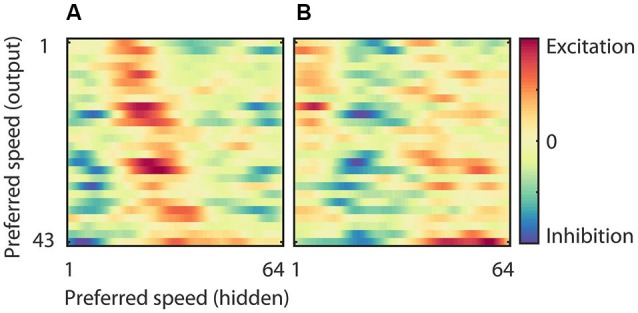
**Weights of the hidden to output units. (A)** Strength of the connection between neurons in the hidden layer and those in the output layer sorted according to the preferred speed of the hidden units (*x*-axis) and the output units (*y*-axis) in the preferred motion direction. This panel shows, for instance, that the output units that preferred high speeds had excitatory connections to hidden units with high preferred speeds, and inhibitory connections to units with low preferred speeds. **(B)** Strength of the connection between neurons in the hidden layer and those in the output layer sorted according to the preferred speed of the hidden units in the anti-preferred motion direction (*x*-axis) and the preferred speed of the output units in the preferred motion direction (*y*-axis). This panel shows that many output units have relatively strong connections to neurons that prefer fast speeds in the anti-preferred direction and are inhibited by neurons that prefer low and intermediate speeds in the anti-preferred direction.

The RMM generated output units with a range of preferred speeds matching our sample of MT neurons. The model allowed us to investigate how this range was constructed from a single population of hidden units. Figure 7 shows the strength of the connection between neurons in the hidden layer and those in the output layer. In panel **(A)** we sorted both the hidden units (*x*-axis) and the output units (*y*-axis) according to their preferred speed for motion in the preferred direction. This shows, not unexpectedly, that output units with, for instance, high preferred speeds had excitatory connections to hidden units with matching high preferred speeds, and inhibitory connections to units with non-matching preferred speeds. Panel **(B)** shows the same connection strengths, but now we sorted the hidden units (*x*-axis) according to the preferred speed for motion in the anti-preferred direction. This shows that many output units had relatively strong connections to neurons that preferred fast speeds in the anti-preferred direction and were inhibited by neurons that preferred low and intermediate speeds in the anti-preferred direction.

This connectivity analysis shows that the motion tuning of the output units arose from the weighted combination of the motion tuning of the hidden layer. They received excitation from hidden units with matching preferred speeds in the preferred direction as well as from hidden units with non-matching preferred speeds in the anti-preferred direction. And, they were inhibited by hidden units with non-matching preferred speeds in the preferred direction as well as by hidden units with matching preferred speeds in the anti-preferred direction. While this provides an intuitive explanation of the motion tuning of the output units, it obviously raises the question how the hidden units generated their motion tuning. We turn to this question next.

### Hidden units: tuning properties

**Figure 8:**
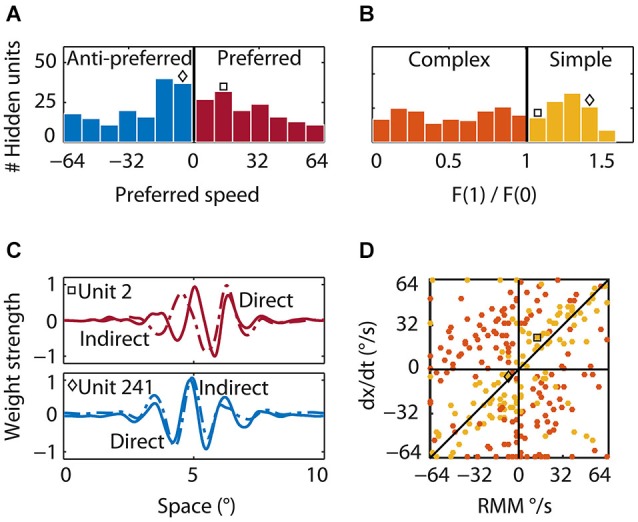
**Properties of the hidden units. (A)** Preferred speed. Roughly half of the hidden units were tuned to the preferred (red bars) and half to the anti-preferred motion direction (blue bars) of the output units. Whereas many units that were tuned to the preferred direction were tuned to the average preferred speed of the output units, many units that were tuned to the anti-preferred direction were tuned to lower than the preferred speed. The square indicates the preferred speed of the first example hidden unit and the diamond the preferred speed of the second example hidden unit in **(B)** and **(C). (B)** Relative response modulation. We classified simple (yellow bars) and complex hidden units (orange bars) according to the conventional cutoff value of F_1_/F_0_ = 1. The square and diamond use the same convention as in **(A). (C)** Direct and indirect inputs for two example hidden units. The first unit (top; square) was tuned to the preferred motion direction of the RMM output units. The direct (solid) and indirect (dashed) input weight distribution were Gabor-like and the indirect input was shifted to the left of the direct input. The second example unit (bottom; diamond) was tuned to the anti-preferred motion direction of the RMM output population and had an indirect peak input shifted to the right of the direct peak input. **(D)** The preferred speed of the simple (yellow) and complex (orange) hidden units as predicted by a linear summation scheme (*y*-axis) plotted against the actual preferred speed (*x*-axis). Positive values show the preferred speed in the preferred direction. Negative values show the preferred speed in the anti-preferred direction. Most simple-like hidden units (like the two example units) were not on the diagonal. This shows that the RMM does not use a motion-energy like linear computation with fixed delays between spatially shifted detectors to compute velocity. This is even more pronounced for the complex-like units where the real preferred motion direction was often opposite to the prediction of the linear summation scheme (opposite quadrants).

We determined direction and speed preference for all hidden units by presenting moving patterns (see Section Materials and Methods) and measuring the hidden units’ tuning curves. The preferred speeds of the hidden units ranged from 1°/s to 64°/s in both the preferred (143 units, mean 30°/s) and anti-preferred (157 units, mean 30°/s) direction of the RMM population response (Figure 8A, red and blue bars, respectively). Many of the hidden units that were tuned to the preferred direction also preferred the same speed as the RMM population. In contrast, many of the hidden units that were tuned to the anti-preferred direction preferred speeds lower than the average preferred speed of the RMM population.

Second, to classify hidden units as simple or complex, we presented sinusoidal gratings, recorded the responses, and determined the ratio of the response modulation at the temporal frequency of the stimulus and the mean response. This is the F1/F0 ratio—a measure often used to categorize simple and complex cells (Movshon et al., 1978a; Dean and Tolhurst, 1983; Skottun et al., 1991). Across the population of hidden units, the distribution of F1/F0 was not bimodal (Figure 8C), suggesting a continuum of simple and complex units, but we used the conventional cutoff (Skottun et al., 1991) of F1/F0 > 1 (see Section Materials and Methods) to classify 124 hidden units as simple-like (yellow bars) and 176 hidden units as complex-like (orange bars).

Third, we investigated how the hidden units were connected to the input and to other hidden units. Many hidden units had Gabor-like input weights, but others had no clear spatial structure. Principal component analysis on the input weights of all the hidden units revealed six components that explained 98% of the variance (data not shown). These eight components could be grouped into three pairs whose input weights were roughly in quadrature, but with increasing spatial frequencies. These weight patterns provide the building blocks that lead to the filters that were extracted with reverse correlation of the output units (Figure 4).

### Hidden units: noise analysis

We used the same white noise analysis previously applied to the output units to gain more insight into the functional properties of the hidden units. First, both simple-like and complex-like units had multiple slanted filters with excitatory and/or suppressive symmetric nonlinearities. Excitatory filters typically corresponded to the preferred speed and direction while suppressive filters corresponded to the anti-preferred speed and direction of the unit, albeit with broader tuning. In other words, their properties were qualitatively similar to those of the output units (Figure 5). Second, the amount of information in the STA for each hidden unit correlated well with the F1/F0 ratio (*r* = 0.78, *p* < 0.001), confirming that simple units had STA’s while complex-units were dominated by STC filters. Finally, the ratio of the amount of information in the first asymmetric filter with the sum over the next twenty symmetric filters varied widely (mean 25, range 5–38). This is in line with our previous finding of a continuous and not bimodal distribution of simple- and complex-like units.

### Hidden units: computing motion through recurrence

Finally, we investigated the feedforward connectivity from the input to the hidden units and the lateral connectivity among hidden units. We defined a hidden unit’s direct weight as the pattern of weights connecting it to all input units. A hidden unit’s indirect weight was defined as the average weight that connected the unit to the input units via the lateral connections of the hidden layer (see Section Materials and Methods). Figure 8C shows the direct (solid) and indirect (dashed) input weights of two example simple-like hidden units, one tuned to the preferred direction (red square) and one tuned to the anti-preferred direction (blue diamond) of the RMM output population. The first example hidden unit had a direct input that was Gabor-like with a peak at 5° and an indirect input that was also Gabor-like, but shifted to the left by 0.5°. For the second example hidden unit, the Gabor-like indirect input was shifted 0.1° to the right of the direct input. This shows that the network self-organized spatially asymmetric recurrent weights; such a connectivity pattern has been shown to generate direction selectivity (Mineiro and Zipser, 1998).

An alternative hypothesis of the mechanism underlying direction selectivity in the RMM starts from the observation that spatially shifted (d*x*) inputs through recurrent connections were always delayed by a single time step (d*t*; the time step of our simulations). This suggests that the hidden units could compute motion in the same way as proposed in the ME model: by the linear summation of two spatially shifted, temporally delayed inputs. Note that by considering only a single time step this view is in fact an inappropriate (non-recurrent) simplification of the recurrent network, even though it may seem a natural simplification within the ME framework. We follow this hypothesis here only because its predictions are informative (but falsified by the data). This scheme predicts that the preferred speed of the units is given by d*x*/d*t*, where d*x* is the spatial shift between the direct and indirect input, and d*t* is the time delay between the direct and the indirect input which was 13 ms in our simulations. In Figure 8D we plot this linear summation prediction (d*x*/d*t*) against the actual preferred speed for all simple-like (yellow) and complex-like (red) units whose spatial shifts could be estimated reliably (see Section Materials and Methods).

The wide scatter of the data points clearly shows that the population as a whole did not follow the prediction based on the ME model. While some simple-like units were relatively close to the slope-1 line, most are not, and the linear summation scheme either overestimated or underestimated the real preferred speed. This mismatch was even more pronounced for most of the complex-like units where, surprisingly, the linear summation scheme often predicted the opposite direction of motion (orange data points in the second and fourth quadrant).

One way to phrase this result is that the recurrent network connectivity changed the effective d*t* from the fixed 13 ms delay generated by the simulation time step, and/or the effective d*x* from the fixed distance between the direct and indirect inputs. Consistent with the view that complex-like units are more driven by the recurrent network dynamics than the simple-like units, which are dominated by the afferent input, these dynamic changes in the spatiotemporal response properties are more pronounced in complex-like units than simple-like units.

In the RMM all hidden units project to the output units. As a consequence, both simple-like and complex-like hidden units contributed equally to the velocity tuning of the output units. As this may appear to conflict with the evidence that MT cells receive mainly V1 complex input (Movshon and Newsome, 1996), we also developed a simple modification of the RMM with two layers of recurrently connected hidden units with feedforward connections between them. This network performed comparably to the RMM studied in detail here, but its first hidden layer developed mainly simple-like units, while the second developed mainly complex-like units. This shows that anatomical constraints can easily be incorporated in the RMM.

## Discussion

We showed that a recurrent network can generate the velocity-tuned response dynamics measured in area MT. This network used only a single intrinsic delay, but nevertheless generated output units with a wide range of speed preferences. When the output units were tested with noise stimuli, they had slanted space-time filters with symmetric nonlinearities for the preferred and anti-preferred direction of motion, much like a feedforward ME network. The RMM, however, captured the full time course of velocity tuning, while the feedforward approximation could not. This strongly suggests that higher than second order spatiotemporal interactions play an important role in motion detection and that they may result from recurrent interactions within the motion network. The hidden units of the RMM showed a continuum of simple- to complex-like properties consistent with those found along the motion pathway of the primate brain. The velocity tuning of these units did not arise from the linear summation of spatially shifted and temporally delayed inputs (as in the ME model), but instead relied on asymmetric spatial connectivity and the nonlinear operations embedded in the recurrent interactions to become sensitive to a wide range of velocities.

After discussing some of the practical limitations of our modeling effort, we discuss the origin of delays in the RMM, the importance of considering the full time course of motion selective responses, and compare the RMM to the ME model.

### Limitations

Our experiments used a stimulus with a diameter of 10° and a monitor refresh of 75 Hz (13 ms). This naturally determines the spatial and temporal bounds on the motion tuning we could find (and then model). For instance, due to aliasing, stimuli moving at 64°/s on a 75 Hz monitor generate limited directional motion signals, hence we did not attempt to model MT neurons with very fast speed preferences. Similarly, very slow movements are affected more by the discretization of space (the limited number of input neurons) and our representation of the random dot patterns removed high spatial frequency and therefore some low speed information. In other words, the particular choices we made to approximate the spatiotemporal properties of the stimuli used in the experiment (e.g., RF size, low-pass filters, simulation time step) limited the range of neurons that the RMM could feasibly model. Our selection of 26 neurons (for instance from the middle of the range of speed preferences) was partially based on that. Hence, we do not claim that the specific RMM used here can model the response of any MT neuron; neurons with very high preferred speed, for instance, would likely require an input layer spanning a larger part of space. Interestingly, this is consistent with the finding that preferred speeds increase with RF size (Orban et al., 1986) and the suggested early stage of speed tuning in the model of Chey et al. (1998).

Our fixed 13 ms simulation time step is a crude abstraction of the dynamics of the visual system. This window was mainly chosen for practical reasons. First, the random dot patterns were displaced every 13 ms; by choosing a simulation time step of (at most) 13 ms we could simulate the response to each pattern that was shown to the neuron. Shorter simulation time steps would have come at rapidly increasing computational cost, but would also require us to use spike count estimates from shorter windows, which would have made these estimates less reliable. Finally, we note that the 13 ms time step is within the approximate temporal integration range of 10–30 ms for pyramidal cells in cortex (Spruston and Johnston, 1992; Trevelyan and Jack, 2002; Zhang, 2004; Léger et al., 2005), hence it does not seem inappropriate to lump activity within such a window. We acknowledge, however, that interesting structure may be found in the time course of motion selective neurons at even shorter time scales.

### Delay lines

The RMM provides a proof of principle that a network, in which all neurons have the same intrinsic delays, can nevertheless generate motion sensitivity with a wide range of preferred speeds. This speed tuning is the result of spatially asymmetric connections (input weights) and nonlinear recurrent dynamics (lateral and recurrent weights) that generate a range of effective delays (Mineiro and Zipser, 1998; Sabatini and Solari, 1999).

A simple linear combination of the spatially asymmetric (d*x*) and the temporally delayed (d*t*) inputs as envisaged in the feedforward ME model did not provide an accurate account of velocity tuning for the hidden units of the RMM (Figure 8D). This reinforces the point that the properties of a single unit in a network dominated by recurrent connections cannot be fully understood on the basis of a snapshot of its input. Moreover, the origin of the temporal delays in the RMM is conceptually different from the feedforward ME model where classes of slow and fast neurons generate speed tuning, or the Reichardt detector with its explicit delay lines. Importantly, the RMM’s mechanism is not contradicted by the finding that the inputs to DS simple cells look as if they originate from fast and slow populations (DeValois and Cottaris, 1998). In the RMM the inputs look like that as well, but because all units have the same intrinsic delay (13 ms; our simulation time step) we know that an interpretation in terms of two populations with different intrinsic properties is incorrect. Hence at the very least our findings serve as a caveat to the interpretation of these empirical findings.

In other recurrent network based motion models (Suarez et al., 1995; Maex and Orban, 1996) temporal delays were implemented by the use of slow (NMDA, GABA-B) and fast (GABA-A, non-NMDA) synaptic transmission. Given that these intrinsic parameters are fixed, this approach can only generate different speed preferences by changing the spatial offset between inputs. This is contradicted by the finding that both spatial and temporal offsets affect the computation of velocity (Koenderink et al., 1985). Our findings (Figure 8) show that a recurrent network does not require a range of intrinsic delays to generate a range of effective delays (and hence speed preferences); it achieves this by the judicious choice of network connectivity strengths. This is in line with Clifford et al. (1997) and Clifford and Langley (2000) who suggested that the temporal filter of the Reichardt detector and the ME model can be implemented recursively and that this significantly reduces computational and storage costs.

Of course our model cannot exclude the possibility that factors other than recurrent network dynamics also contribute to the computation of velocity. For instance, variations in the temporal properties of the thalamic relay neurons that provide input to DS cells in primary visual cortex can contribute to direction selectivity (Saul, 2008). Such a mechanism is likely more important in species such as the cat where separate populations of lagged and non-lagged thalamic relay cells are well-established (Mastronarde, 1987). In our view, the strength of data-driven modeling approaches is that they, unlike experimental work, can isolate potential contributing factors (e.g., recurrent dynamics, intrinsic delays, receptor kinetics). This generates a better understanding of the underlying computational principles, even when the factors likely interact in the biological network.

### Time course

The time course of velocity tuned responses has received little attention in models, but can be quite revealing of the underlying mechanisms. Our analysis in Figures 6C,D, for instance, shows that the best feedforward LN-model, unlike the RMM, cannot capture the full velocity tuning properties of the MT cells. The same is true for the implementation of the ME model by Simoncelli and Heeger (1998) which considers only steady-state velocity tuning. Because both the LN approximation to the RMM and the ME model rely only on spatiotemporal correlations up to second order, this mismatch strongly suggests that MT neurons are driven by higher-order spatiotemporal correlations. This provides a novel insight into the phenomenon of sequential recruitment: motion selectivity in MT (Mikami, 1992) and motion detection performance (McKee and Welch, 1985) improves nonlinearly with the number of successive steps in an apparent motion sequence. Our model suggests that this phenomenon relies critically on the recurrent network dynamics.

### Comparison to the motion energy model

When analyzed with white noise methods, the RMM revealed filters and nonlinearities that were at least superficially consistent with the ME model (i.e., slanted in space time, a quadratic nonlinearity, and a form of motion opponency). While this provides support for the model (because such filters have been found empirically), it also makes an important conceptual point about the interpretation of the empirical data. Notably, finding such ME-like filters in real neurons does not prove that the underlying architecture is at all similar to the feedforward ME model.

We also found a number of deviations between the RMM and the idealized ME model (Adelson and Bergen, 1985); in each case the RMM properties are supported by the empirical data. First, reverse correlation of the output units as well as that of the hidden units revealed many more than four space-time filters. This is supported by the large number of slanted space-time filters in V1 simple and complex cells (Rust et al., 2005). The RMM also deviates from the idealized ME model in its use of motion opponency. The filters of the output and hidden units, for instance, were not mirror opposites of each other (Figure 4B) as would be expected from pure motion opponency. This asymmetry can also be seen in the speed tuning curves of the MT neurons (Figure 3A), and it is compatible with the DS V1 cells from Rust et al. (2005) and our previous finding that motion opponency in MT involves a competition among multiple Fourier components, rather than a strict inhibition between opposite velocities (Krekelberg and Albright, 2005).

## Conclusion

A recurrent network can compute a representation of velocity in much the same way as the ME model, but without the need for separate classes of fast and slow neurons or synapses. In contrast to the ME model, the recurrent network also matches the temporal dynamics of a population of single MT cells, and makes use of higher-order spatiotemporal correlations in the input. Because it relies on the pervasive recurrent connections of visual cortex, and given that it contains hidden units that are similar to other neurons in the motion processing pathway of the primate brain, we believe it is a biologically plausible model of motion detection.

Even though we focused on motion detection here, the training of artificial recurrent networks on recorded neuronal responses may also be a generally useful approach to investigate other domains of sensory processing and higher cognitive function that require the representation of sequences and time, which is thought to depend critically on recurrent network dynamics (Elman, 1990).

## Conflict of interest statement

The authors declare that the research was conducted in the absence of any commercial or financial relationships that could be construed as a potential conflict of interest.
